# EFL Students' Preferences for Written Corrective Feedback: Do Error Types, Language Proficiency, and Foreign Language Enjoyment Matter?

**DOI:** 10.3389/fpsyg.2021.660564

**Published:** 2021-04-08

**Authors:** Tiefu Zhang, Xuemei Chen, Jiehui Hu, Pattarapon Ketwan

**Affiliations:** ^1^School of Foreign Languages, University of Electronic Science and Technology of China, Chengdu, China; ^2^Martin De Tours School of Management and Economics, Assumption University, Bangkok, Thailand

**Keywords:** written corrective feedback, Foreign language learning, english as a Foreign language, Foreign language enjoyment, learner preference, learner proficiency

## Abstract

Using both quantitative and qualitative approaches, this study investigated the preference of learners of English as a foreign language (EFL) for four types of written corrective feedback (WCF), which are often discussed in the literature, on grammatical, lexical, orthographic, and pragmatic errors. In particular, it concerned whether such preference is influenced by two learner variables, namely, foreign language enjoyment (FLE) and proficiency level. The preference for selective vs. comprehensive WCF was also examined. The participants in the study were 117 University students in a Thai EFL context. Analysis of questionnaire data revealed a tendency for learners to prefer more explicit types of WCF (i.e., metalinguistic explanation and overt correction) for most error types, irrespective of their proficiency and FLE level. High proficiency level learners rated less explicit WCF types (i.e., underlining and error code) as useful to some degree, whereas their low proficiency level counterparts did not. Similar results were found for the two FLE groups. Besides, the FLE level seemed to play a role in perceiving the value of WCF in terms of scope. The results of follow-up interviews showed that the linguistic features of learners' first language, existing knowledge of the target language, affective feelings, and teacher's role were the main factors contributing to variation in learners' preferences. Possible pedagogical implications are discussed.

## Introduction

Written corrective feedback (WCF) is an important aspect of second or foreign language pedagogy and has been extensively researched in the field of both second language acquisition (SLA) and second language (L2) writing. WCF occurs in response to linguistic errors made in learners' writing (Bitchener and Ferris, 2012). On most occasions, WCF is provided by teachers, peers, or computers in classrooms, but it sometimes can be provided by native speakers and non-native learners in naturalistic settings. In this study, we focus on teacher WCF as it is the most common practice that has been of great interest to teachers and researchers in English as a foreign language (EFL) contexts. Since the debate initiated by Truscott (1996) on the effectiveness of WCF, there has been increasing evidence that WCF can facilitate improved accuracy in subsequent writing, immediately and over time (for meta-analyses, see Kang and Han, 2015; Lim and Renandya, 2020). However, such effect appears to be linked to linguistic factors (e.g., feedback or error types) and depends upon learners' perceptions of WCF, particularly their preferences for WCF types, and affective factors such as positive emotions.

Research on WCF has identified different WCF types provided by teachers in the classroom (Ellis, 2009). Indirect WCF appears to be the most frequently used feedback type in the classroom; however, it seems not to be preferred by lower proficiency learners (e.g., Lee, 2008; Amrhein and Nassaji, 2010; Chen et al., 2016). There is also evidence that learners have a higher preference for WCF on grammatical errors than lexical or mechanical errors, irrespective of proficiency level (Zhang, 2018). These findings may suggest that (1) learners may not benefit from WCF if their preference of it is discrepant from teachers' practices (Rummel and Bitchener, 2015), and (2) the preference is related to error types and proficiency level. Thus, exploring learners' preference of WCF type helps gain a better understanding of the role of WCF in L2 learning, and it is necessary to consider the impact of proficiency level and error types on learners' preferred WCF types.

Moreover, affective factors are recognized as being important in understanding learners' actions in L2 learning activities (Dörnyei, 2005). This also applies to the instruction of WCF. Learner affective engagement with teacher WCF can affect how learners perceive and respond to the WCF they receive (Ellis, 2010). For instance, teachers are suggested to correct a certain number of errors at a time to minimize learners' negative emotions (Lee, 2019); however, learners tend to prefer to have all errors corrected (e.g., Lee, 2005; Alshahrani and Storch, 2014). Therefore, investigating learners' emotions may help explain their preference for a WCF type.

Given that an adequate discussion of the effect of emotions on WCF is notably missing from WCF studies (Bitchener and Storch, 2016), this study aims to investigate further learners' WCF preferences concerning their emotional states. More specifically, it focuses on one discrete type of emotions, namely, foreign language enjoyment (FLE). WCF, as a form of negative evidence, is often related to negative emotions such as anxiety (Yu et al., 2020), whereas positive emotions like enjoyment is also experienced by learners in WCF situations (Han and Hyland, 2019), and to date, little is known about its influence on learners' preferences for WCF types.

## Literature Review

### Written Corrective Feedback Types and Their Role in Second Language Development

Much recent WCF research has been cognitively oriented, motivated partly by practical interest to understand how it can be most effectively provided (Bitchener and Ferris, 2012). This line of inquiry helps inform teachers on what types of WCF they can use to optimize learning outcome and the extent to which they should respond to errors made by learners.

Three broad types of WCF have been identified in the literature: direct, indirect, and metalinguistic (Ellis, 2009). Direct WCF refers to locating errors and supplying correct forms; in some instances, it is in the form of crossing out unnecessary words or adding omitted words. When the presence of errors is identified by underlining but no correct forms are provided, such WCF is indirect. Metalinguistic WCF has similar features as indirect WCF, as it also concerns the error location, withholds the correct form, and promotes self-correction; however, these two types of WCF “are fundamentally different” (Li and Vuono, 2019, p. 100) in that the former provides additional information about the cause and nature of the error. Metalinguistic WCF can be further operationalized by using an error code to indicate the error type or providing a brief metalinguistic explanation about the error. Thus, four types of WCF, namely, overt correction (i.e., direct WCF), underlining (i.e., indirect WCF), error code, and metalinguistic explanation (i.e., metalinguistic WCF), which differ in their degree of explicitness, are the focus of this study. In light of Lee's (2017) classification of WCF types, overt correction is the most explicit type of WCF, while underlining is the least explicit type of WCF. In metalinguistic WCF, metalinguistic explanation is more explicit than error code.

There is growing interest in whether more explicit types of WCF can contribute more to learners' L2 development (e.g., Bitchener and Knoch, 2010; Shintani et al., 2014; Guo and Barrot, 2019). Theoretically, the computational model of Gass (1997) has explained that for learning to take place, learners need first to attend to the target feature and notice the mismatch between the WCF input and their output. They then need to understand the WCF input that has been noticed. WCF is inevitably explicit in nature (Shintani and Ellis, 2013) to function as a noticing facilitator; however, the degree of explicitness of WCF affects the noticing of it. More explicit types of WCF, such as overt correction, may lead to a higher level of noticing.

Moreover, the type of WCF may play a role in understanding errors. The more linguistic information provided in the WCF, the better understanding of the error can be obtained (Sheen, 2007). Drawing on the skill acquisition theory (DeKeyser, 2007), it is understandable that the proceduralization of L2 knowledge can occur if learners have opportunities to use the knowledge through practice. Thus, one would expect that error code and metalinguistic explanation are beneficial in that (1) they enable learners to understand the nature of the error, and (2) they provide learners with opportunities to self-correct, thereby facilitating reflection on partially acquired knowledge of an L2. Therefore, it is worth considering the way learners perceive the received WCF due to its explicitness of error correction and the way they respond and process.

In addition to the above classification, according to Liu and Brown (2015), WCF can be categorized into three types in terms of scope: highly focused (only one error type is targeted), mid-focused (multiple error types, e.g., two to six), and highly unfocused (all errors). The first two approaches are referred to as selective WCF while the last one as comprehensive WCF (see Mao and Lee, 2020 for a review). SLA researchers have been primarily concerned with highly focused WCF in relation to theoretical claims about the importance of, as Schmidt suggests, “attention” and “understanding” (Schmidt, 2001). Highly unfocused WCF is postulated to be less effective than highly focused WCF because it can easily lead to an overload of information processing (Bitchener, 2008; Sheen et al., 2009). However, the highly focused WCF is criticized for its lack of ecological validity and pedagogical significance for real classrooms (Storch, 2010), given that learners generally expect an overall accuracy improvement by having all errors corrected (e.g., Lee, 2005; Amrhein and Nassaji, 2010). In solving this dilemma, Lee (2019) suggests that teachers adopt a mid-focused approach to WCF as a compromise. Research shows that resistance to WCF may occur when learners see little value in it (e.g., Storch and Wigglesworth, 2010); thus, it is necessary to examine how learners perceive the usefulness of selective and comprehensive WCF in order to provide implications for teachers in justifying WCF instruction.

### Effect of Error Type on Written Corrective Feedback

Recent studies have revealed that learners' perceptions and responses to WCF seem to be affected by error types. For example, Hanaoka (2007) showed that WCF was more likely to direct the attention of Japanese learners of English to lexical errors but not to other types of errors. Similar findings were observed in the study of García Mayo and Labandibar (2017) conducted with Spanish learners of English. In the study of Simard et al. (2015), although learners could notice WCF triggered by lexical errors, they sometimes wrongly interpreted teachers' intention of correcting word choice errors as spelling errors. In contrast, Zhang (2018) reported that it was easier for EFL learners to perceive WCF on orthographic errors than lexical errors. Moreover, many of the learners felt confused by WCF provided on pragmatic errors (e.g., the incorrect forms of politeness), which are pragmatically inappropriate but grammatically correct; they could only guess about the possible reason for the correction, as explained by Schauer (2006), probably due to their lack of awareness of pragmatic infelicities. It has been suggested that the more explicit the WCF is, the more the accurate understanding of errors is likely to be (Stefanou and Révész, 2015; Suzuki et al., 2019).

Truscott (1996) has argued that, theoretically, no single form of WCF can be expected to help learners address all types of errors. His concern is confirmed by Van Beuningen's et al. (2012) investigation revealing that learners of Dutch's grammatical accuracy benefited only from direct WCF, which, however, led to less non-grammatical accuracy gains than indirect WCF. One interesting finding of survey studies (e.g., Amrhein and Nassaji, 2010) was that learners generally considered WCF to be most useful for grammatical errors, followed by lexical errors, spelling errors, and punctuation errors^1^. It appears that learners' interpretation of, and reaction to, teachers' WCF on different error types are associated with the characteristics of linguistic aspects. Therefore, in addition to grammatical errors that frequently occurred in student writing, the present study also investigates WCF types with respect to non-grammatical errors, including lexical, orthographic, and pragmatic errors.

### Learners' Preferences for Written Corrective Feedback, Proficiency Level, and Foreign Language Enjoyment

As the above review reveals, the effectiveness of WCF is related to learners' understanding of, and reaction to, WCF types on different errors. Positive attitudes held by learners toward WCF indicate their need for improvement in writing performance (Chen et al., 2016), and mismatched perceptions of WCF between learners and teachers is likely to impede learners' ability to use WCF effectively (Zhang, 2018; Saeli and Cheng, 2019). The study of Zhang (2018) further demonstrated that learners' awareness of teachers' feedback intention might influence how they perceive the effectiveness of a particular WCF type. These studies highlight the importance for teachers to consider the preferences of learners when providing WCF.

Previous studies have shown that learners have a strong preference for WCF. A handful of experimental studies have attempted to explore learners' favorite WCF types (e.g., Diab, 2015; Rummel and Bitchener, 2015). One tension in these studies concerned teachers' relative emphasis on grammatical errors in student writing. Diab (2015) found that learners preferred error code to overt correction for lexical errors. In the study of Rummel and Bitchener (2015), investigating the comparative effectiveness of direct vs. indirect WCF on grammatical accuracy, most learners preferred to receive the latter (i.e., underlining). What is missing from these studies is a consideration of learners' perceptions of WCF provided for pragmatic errors. While understanding of how learners perceive the role of oral corrective feedback (OCF) such as recasts on pragmatic is growing (e.g., Yoshida, 2010; Yang, 2016), this area is underexplored for WCF research.

Another factor that may influence learners' preference for receiving WCF is their proficiency level. A survey study by Chen et al. (2016) indicated that perceptions of WCF on grammatical errors varied among Chinese EFL learners with different proficiency levels: error code was preferred by intermediate learners, and overt correction was preferred by advanced learners. However, these findings seem not to help explain the claims that less explicit WCF, such as error code, benefits higher proficiency learners as it allows them to “engage in guided learning and problem solving” (Bitchener and Knoch, 2008, p. 415). In contrast, more explicit WCF, such as overt correction, suits lower proficiency learners' needs as it is “more immediate” and helpful for successful self-correction (Bitchener and Ferris, 2012). In terms of the distinction between selective and comprehensive WCF, SLA researchers (e.g., Sheen, 2007; Bitchener, 2008) suggested that lower proficiency learners may benefit more from highly or mid-focused WCF due to their limited attentional resources. However, both intermediate and advanced learners in Chen et al. (2016) favored a selective WCF approach. Thus, learners' proficiency level needs to be considered when investigating their preferences for WCF.

Apart from proficiency level, the ongoing “affective turn” highlighting the role of emotion in SLA (Pavlenko, 2013, p. 5) indicates that learners' affective factors are also likely to exert influence on learners' preference for WCF. Foreign language anxiety, for example, has been found to be linked to different types of error correction. Specifically, research on OCF found that low-anxiety learners benefited most from metalinguistic explanation (similar to the one provided in the written context), whereas high-anxiety learners benefited most from recasts (Rassaei, 2015). Likewise, the influences of Positive Psychology on SLA research indicate that positive emotions like FLE may also potentially affect learners' gains from different types of error correction. While the literature has largely considered the effects of negative emotions, the effects of positive emotions on learners' L2 development with regard to different types of error correction has, to date, hardly been discussed.

Drawing on the broaden-and-build theory (Fredrickson, 2001), positive emotions such as FLE is crucial in SLA as it boosts learners' capacity to notice things in language input, increases learners' resilience and hardiness in inevitable moments of struggle, and has longer-term consequences in classrooms (see Dewaele et al., 2019 for a review). Enjoyment, as Boudreau et al. (2018) defined, takes on dimensions such as intellectual focus, heightened attention, and optimal challenge, and is a powerful motivator in SLA (Dewaele and Alfawzan, 2018). It depends on interactions with teachers, peers, and classroom activities (Dewaele and MacIntyre, 2014) and has a dynamic and complex relationship with foreign language anxiety (Dewaele and MacIntyre, 2016). To measure FLE, a 21-item FLE scale with two dimensions—social FLE and private FLE—was developed by Dewaele and MacIntyre (2016). Social FLE is reflected by good relationships with teachers and peers, while private FLE is associated with learners' pride in achievement, especially at something difficult and fun in L2 learning.

Previous studies have found variation in FLE as a function of gender difference (Dewaele et al., 2016), teacher and teacher practice (Dewaele et al., 2018), other teacher-centered variables such as attitudes toward the teacher, friendliness of the teacher, and joking by the teacher (Dewaele and MacIntyre, 2019), and learner-internal variables such as attitudes toward the target language and proficiency level (Dewaele et al., 2018). Specifically, Dewaele et al. (2018) found that intermediate learners were reported to have more FLE than low intermediate learners. FLE has also been demonstrated to be a strong predictor of learners' willingness to communicate (Dewaele and Dewaele, 2018), learners' performances on lexical decision tasks (Dewaele and Alfawzan, 2018) and L2 achievement (Jin and Zhang, 2018). Moreover, FLE is found to have a mediating role in the relationships between learners' motivation and language proficiency (Zhang et al., 2020). Taken together, FLE is most likely to be triggered by teacher-related factors, boosts learners' capacity in FL, affects the process of the target language, and mediates other affective factors such as motivation as well as learners' proficiency level. Given the important role of FLE in L2 learning, the present study probes into the predictive value of FLE for learners' WCF preferences.

## Methods

The present study is part of a larger investigation (Zhang, 2018) of the role of WCF for L2 development in relation to individual difference variables. To advance our knowledge of learners' preferences of WCF on different error types concerning their proficiency and FLE level, this study aims to address the following research questions:

1. What are EFL learners' preferences for WCF on different error types?
2. Do EFL learners' proficiency and FLE level affect their preferences for WCF types?
3. Why do EFL learners prefer certain types of WCF for specific errors?

To address the first two research questions, a questionnaire was conducted to collect information on learners' background characteristics (age, gender, English proficiency level, and learning experience). For the third question, data were collected mainly through semi-structured interviews. The qualitative findings from the interviews would also allow us to better understand the quantitative findings from the questionnaire stage (Creswell, 2014).

### Setting

The particular context of the study is an English for academic purposes (EAP) program at a large Thai University using English as the medium of instruction. The language department of the university offered a range of English courses for local students to develop their academic English language skills and cultural awareness. Table 1 presents the program structure, which suggests, for example, that 120 instruction hours of English I study was the rough equivalent of IELTS band 5^2^.

**Table 1:** English program structure and IELTS band.

**English course**	**Instruction hours**	**Equivalent IELTS scores**
Basic English	200	5.0 below
English I	120	5.0
English II	120	5.5
English III	120	6.0
English IV	120	6.5

The basic course was designed to lay the foundation for preparing students to undertake the main courses. As EAP was a compulsory course at the university, students enrolled in the program were required to attend three academic English classes and one conversation class weekly. Writing was a key part of the EAP curriculum, aiming to foster English skills through communicative activities in a meaningful academic context. In particular, students were trained to recognize and correct their written production errors when completing English IV.

### Participants

For this study, non-probability purposive sampling was used to recruit first year students from the EAP program as research participants^3^. A total of 156 students agreed to participate in the study and completed two surveys (see the *Instruments* section for details): a WCF preference questionnaire (WCFPQ) and an FLE scale (FLES). According to participants' scores on the FLES, two subgroups (low and high FLE) were formed using a third-split method as in previous studies (e.g., Sheen, 2008; Zhang and Rahimi, 2014). Low FLE students (*n* = 56) were defined as scoring more than one standard deviation below the mean and high FLE students (*n* = 61) as scoring more than one standard deviation above (the total mean and standard deviation for the whole sample are 3.72 and 0.59, respectively). It should be noted that students (*n* = 39) were excluded in the study if their mean scores fell within one standard deviation of the mean on the FLES.

As Table 2 shows, the final sample included 117 students whose English proficiency level, as measured by the university placement test, ranged from low to upper immediate. All the students from basic course classes (*n* = 55; 25 females, 30 males) were considered low intermediate; the rest of the students (*n* = 62; 33 females, 29 males) had completed English II, demonstrating that they achieved an upper intermediate level. These students were between 18 and 23 years of age and came from various majors, including engineering, laws, arts, architecture and design, and economics. Additionally, none of the students learned additional foreign languages other than English, except nine participants exposed to Chinese in their early years of schooling.

**Table 2:** Participants' information.

	**Low intermediate**	**Upper intermediate**
Number	55	62
Age	Range = 18–20	Range = 19–23
Gender	25 (Female); 30 (Male)	33 (Female); 29 (Male)

Note that the surveys were administered at the end of the semester due to several logistic constraints, which did not allow for the recruitment of many interview participants. For each proficiency level, four students—two had low FLE and two high FLE—were invited voluntarily for the follow-up interview, making a total of eight interviewees Thus, the interview sample was small but involved both proficiency (low and upper) and FLE (low and high) levels, which possibly helped obtain an overview of participants' WCF preferences. More information about the interview participants was provided in Table 3.

**Table 3:** Interviewees' backgrounds.

**No**.	**Age**	**Pseudonym**	**Gender**	**Course**	**FLE level**	**Major**
1	18	Tong	Male	Basic	Low	Laws
2	18	Tiw	Male	Basic	High	Engineering
3	19	Aui	Female	IV	High	Engineering
4	21	Diow	Female	IV	High	Arts
5	19	Swattia	Female	Basic	Low	Economics
6	18	Dan	Male	Basic	High	Laws
7	20	Pat	Female	III	Low	Arts
8	20	Wanlada	Female	IV	Low	Arts

### Instruments

#### The Written Corrective Feedback Preference Questionnaire

Data on participants' preferences for WCF types for different error types were collected using a WCF preference inventory (see Appendix 1 in Supplementary Material). The questionnaire was initially written in English and then translated into a Thai version by an independent English–Thai translation expert. To ensure the two versions were equivalent, two experienced EFL Thai teachers were invited to comment on the naturalness of the translation and the content of the questionnaire to fine-tune it. The questionnaire was divided into three sections. Section A contained demographic questions about students' age, gender, L1 background, foreign language learning experience, and the courses they were studying. Section B and C comprised 33 closed-ended items concerning explicitness and scope in WCF types preferred by students.

Considering that students might have difficulty interpreting technical terms used for the WCF types, we adopted a method used by previous studies (e.g., Yang, 2016) to design the Section B items. That is, any use of technical terms that refer to WCF types was avoided in the questionnaire; instead, examples of WCF types provided on specific errors were used as questionnaire items. As discussed in the literature review section, investigations of WCF need to consider the ecological validity of findings for the classroom (Storch, 2010). Bearing this suggestion in mind, all the error examples were created based on analyzing samples produced by about half of the students in their classroom writing activities. In Section B, each student was asked to rate the usefulness of four WCF types (i.e., overt correction, underlining, error code, and metalinguistic explanation) on four types of linguistic errors (i.e., grammatical, lexical, orthographic, and pragmatic). For each linguistic error type, two specific error examples were provided accordingly^4^ (for details, see Table 4). The Section C items concerned the extent to which students preferred to be corrected, with one questionnaire item for one particular approach to WCF (e.g., highly focused). All the questionnaire items were structured using a five-point Likert scale, from 1 for *not at all useful* to 5 for *extremely useful* (Section B) or from 1 for *strongly disagree* to 5 for *strongly agree* (Section C). A Cronbach's alpha of 0.81 was obtained for the total questionnaire indicating acceptable reliability.

**Table 4:** Error examples provided in the written corrective feedback preference questionnaire (WCFPQ).

**Linguistic aspect**	**Error aspect**	**Example**
Grammar	(1) English article	There is *the* park near my home
	(2) Regular past tense	I *droped* them into a bag
Lexicon	(1) Word choice	That is our *respectful* teacher
	(2) Redundancy	*All kinds of types of* colors are beautiful
Orthography	(1) Capitalization	Visitors can get to the *grand palace* by boat
	(2) Punctuation marks	Apple will announce *it's* new iWatch
Pragmatics	(1) Requests	I *want* your answer to my question
	(2) Politeness	*Can* you provide any advice for me?

#### The Foreign Language Enjoyment Scale

For measurement of FLE, the researchers adapted the questionnaire developed by Dewaele and MacIntyre (2016). The FLES contained a total of 21 items to which participants responded on a five-point Likert scale, ranging from “strongly disagree” to “strongly agree.” The wordings of several items were slightly modified to be appropriate for the present study. For example, the original item “I can be creative” was changed to “I feel I can be creative in learning English.” The Cronbach's alpha for the total scale was 0.848, indicating high reliability of the instrument. It was also examined whether there was a significant improvement in reliability by removing any of the items (see Appendix 2 in Supplementary Material). Since no such item was found, all the items were kept in the scale. Responses were scored by first calculating the total score for each participant and then dividing by 21. Accordingly, each participant received an average score on a scale of 1–5; a higher score on the scale corresponds to more FLE.

### Semi-structured Interviews

A semi-structured interview was conducted with students (*n* = 8) online and auto-recorded using Zoom. During the interview, students were encouraged to use English but allowed to speak in Thai or mix the two languages to make them feel comfortable and freely express their ideas. The interviews were informal, lasting 15–20 min each. The interview questions were primarily designed to learn about the sources of their preferences for WCF types, some of which invited them to reflect on their responses to the WCFPQ, and others elicited their opinions about preferred WCF types, language proficiency, and FLE level. The interview guidelines used here are as follows:

1. What WCF type students preferred to receive on different error examples presented in the questionnaire, and why they preferred that particular type;
2. When receiving WCF from teachers in actual classrooms, what aspects they liked most and disliked and why;
3. Whether they considered that proficiency was related to preferred WCF types, and why they did or did not;
4. Whether they considered that FLE was related to preferred WCF types, and why they did or did not.

### Procedure and Data Analysis

The study included three phases: (1) FLE phase, (2) WCFPQ phase, and (3) interview phase. This study was approved and supported by the English department of the participating university, and before its commencement, the consent form was signed and returned by all participants. As has been mentioned above, the FLES was initially administered online to a total of 148 students. Then 117 of them were assigned to low and high FLE subsamples based on their scores on the FLES, and they were asked to complete the WCFPQ. Finally, eight students with different FLEs and proficiency levels were involved in the interview phase of the data collection.

To answer the first research question, quantitative data collected from the WCFPQ were analyzed by descriptive statistics using SPSS version 27. In addressing the second research question, a two-way MANOVA was run for each specific error type, with two independent variables of FLE (two levels: low and high) and proficiency (two levels: low and upper intermediate); scores of perceived effectiveness of the four types of WCF as measured by the WCFPQ was considered as the dependent variable. In the case of the data of Section C, a separate two-way ANOVA was used to reveal whether there were any significant results. To answer the last research question, the interview data were transcribed, coded, and analyzed thematically with NVivo 11 following Braun and Clarke's (2006) guidelines.

## Results

### Findings of RQ1

On the 16 items in Section B of the WCFPQ, all participants were asked to rate how useful the four types of WCF that differ in terms of explicitness in treating grammatical, lexical, orthographic, and pragmatic errors are. Table 5 presents the mean scores of ratings on a five-point Likert scale (1 = not at all useful, 2 = not very useful, 3 = somewhat useful, 4 = very useful, 5 = extremely useful).

**Table 5:** Descriptive statistics of rating scores for the WCF types on specific errors.

**Error type**	**Overt**	**Underlining**	**Error**	**Metalinguistic**
	**correction**		**code**	**explanation**
	**M (SD)**	**M (SD)**	**M (SD)**	**M (SD)**
Grammatical	4.02 (0.85)	2.75 (1.19)	3.44 (1.04)	4.52 (0.64)
Lexical	4.35 (0.67)	2.79 (1.13)	3.08 (1.08)	4.06 (0.98)
Orthographic	4.13 (0.86)	2.81 (1.12)	3.31 (1.06)	4.33 (0.92)
Pragmatic	3.29 (1.01)	3.15 (1.07)	3.25 (0.99)	4.25 (0.79)

As the table shows, both metalinguistic explanation and overt correction were highly preferred by students to correct all the four types of errors, with mean scores greater than 4 (with one exception of overt correction on pragmatic errors), respectively. Moreover, students rated metalinguistic explanation as most useful for grammatical (*M* = 4.52), orthographic (*M* = 4.33), and pragmatic errors (*M* = 4.25); overt correction (*M* = 4.35) was most preferred for lexical errors. In contrast, the least preferred WCF type was underlining as the mean scores were lower than 2.82 on all error types except pragmatic (*M* = 3.15). This suggests that students were generally ambivalent about the effectiveness of underlining on grammatical, lexical, and orthographic errors, and held a weak perception of its role for pragmatic errors. As for error code, its usefulness was rated between mean scores of 3.05 to 3.45, indicating that participants tended to view it as a somewhat useful WCF strategy on all types of errors. These results reveal a tendency for the participants to prefer more explicit WCF types regardless of error types.

Section C provided information about participants' preference for the scope of WCF. Students were asked to rate their agreement with the statement “Teacher should correct all errors in student writing, major and minor.” The mixed responses (*M* = 3.59) suggest that students had a neutral preference for comprehensive WCF. In other words, selective WCF appeared to be valued by some of them.

### Findings of RQ2

The effects of proficiency and FLE level on participants' preferred WCF type for each specific error type were assessed using two-way MANOVA. In terms of grammatical errors, there was only a significant main effect of proficiency level on underlining, *F*_(1, 113)_ = 5.73, *p* = 0.02, and on error code, *F*_(1, 113)_ = 4.27, *p* = 0.04. The mean scores of perceived effectiveness of underlining were 2.27 and 3.23 for low and upper intermediate learners, respectively, indicating that this WCF type was negatively evaluated by the lower level group. Similar results were found for error code, with the upper intermediate group (*M* = 3.76) rating higher than the low intermediate level group (*M* = 3.11).

In terms of lexical errors, the MANOVA only showed a significant main effect of proficiency level on error code, *F*_(1, 113)_ = 5.22, *p* = 0.02. Significance was also found for the interaction of proficiency level and FLE level on this type of WCF, *F*_(1, 113)_ = 4.88, *p* = 0.03. The results revealed that upper intermediate learners (*M* = 3.52) tended to be optimistic about the role of error code in treating lexical errors, but it was not the case for their low intermediate counterparts (*M* = 2.64). It was also found, as the significant interactive effect of the two variables suggested, that among all subgroups, low intermediate learners with high FLE showed a higher estimate of effectiveness for error code compared with those with low FLE, as indicated by the mean scores of 2.87 vs. 1.65.

Regarding the perceived effectiveness of WCF on orthographic errors, only the main effect of proficiency level was found on underlining *F*_(1, 113)_ = 6.31, *p* = 0.01. Significance was also observed between low and high FLE groups, *F*_(1, 113)_ = 5.08, *p* = 0.03. The subgroup analysis showed that this type of WCF was considered more useful by the upper intermediate group (*M* = 3.30) than the low intermediate group (*M* = 2.32). Similarly, learners with high FLE tended to view underlining (*M* = 3.11) as more useful than did learners with low FLE (*M* = 2.51).

In terms of pragmatic errors, there was neither any main effect of proficiency or FLE level nor interaction effect of the two variables on the four types of WCF, all *Fs*(1, 113) <2.10, *p* < 0.05. The results reveal that similar perceptions of the effectiveness of specific WCF types were shared by most participants, regardless of their proficiency and FLE level.

The ANOVA results indicated a nonsignificant interaction on participants' perceptions of the usefulness of comprehensive approach to WCF, *F*_(1, 113)_ = 1.16, *p* = 0.28 and the main effect of proficiency level, *F*_(1, 113)_ = 0.86, *p* = 0.36; however, there was a significant main effect of FLE level, *F*_(1, 113)_ = 4.43, *p* = 0.04. Comprehensive WCF seemed more likely to be preferred by low FLE than high FLE learners (*M* = 3.99; *M* = 3.18).

### Findings of RQ3

The interview data were thematically analyzed using NVivo 11 to explore possible reasons for quantitative findings. The five themes that emerged from the qualitative analysis include L1 linguistic features, existing knowledge, affective feelings, and teacher's role. It appears that both proficiency and FLE level are reflected in the themes. The first two themes are related to learners' proficiency level while the other two to FLE level.

Consideration of linguistic features was found to contribute most to participants' preference to a particular WCF type. The participants reported that they regarded grammar as the most important aspect of writing, but most of them found it difficult to solve their grammar-related problems in writing on their own due to confusion of grammar rules. For example, metalinguistic explanation was considered most helpful for grammatical errors mainly because:

“There are no articles in my mother tongue, so I usually neglect using them. I think that English article rules are very complicated, and sometimes even I do not neglect, and I still make errors in writing. It would be helpful if my teacher would explain the rules to me.” (Tiw)

The participants, especially those at a low intermediate level, also considered overt correction useful for learning; yet, when comparing overt correction with metalinguistic explanation, it seemed that they preferred the latter as it allowed them to figure out by themselves the correction of some errors they had previously learned:

“The feedback [overt correction] can also help me revise, but I want to learn more from the error [.] when the cause is explained, I have a chance to think about the correct answer on my own.” (Tong)

Existing L2 knowledge was another important concern. All participants mentioned that they had limited knowledge of English pragmatic conventions, and for this reason, they rated highly on metalinguistic explanation but viewed underlining as ineffective when dealing with pragmatic errors. The following excerpt demonstrates this:

“It is hard for me to deal with pragmatic errors of such as politeness even if teachers point them out because I know very little about what can be considered acceptable or unacceptable. With the explanation like this, I could see what was wrong with the [example] sentence.” (Pat)

Diow from the upper intermediate group simply stated, “If I do not know the difference between “want” and “would like,” I may still make the same mistakes when writing emails to teachers.” Also, three low intermediate students considered overt correction better than other three WCF types when dealing with lexical errors, mainly due to their lack of vocabulary knowledge of English.

Another theme—affective feelings—refers to how learners felt about the WCF provided. When talking about metalinguistic explanation, most students showed an increase in motivation to learn from errors, as Wanlada stated: “This feedback would make me think about my errors and want to learn more about grammar.” Another positive comment comes from three high FLE students who would be appreciative of teachers' effort to deliver metalinguistic explanations, making them feel cared for. Another interesting finding is that two of the three students with high FLE commented negatively on comprehensive WCF as they preferred to self-correct some errors:

“I prefer teachers to leave some errors to me and let me try. Perhaps, some students want teachers to correct all errors, but this can be a source of frustration for me.” (Wanlada).

The teacher's role is specifically related to the preference for selective vs. comprehensive approach to WCF. Of the five students (including all the high FLE ones) who responded neutrally or reported their disagreement, many explained that correcting all errors would increase the teacher's workload. In contrast, the other two students felt that it is the teacher's responsibility to correct all errors. Additionally, most of the students noted that they mainly received underlining from teachers but rarely experienced metalinguistic explanation. It was also found that three out of the four upper intermediate learners, who rated error code as “somewhat useful” or above, reported that their teachers frequently use error code. As Walanda noted, “My teacher also uses similar symbols to mark my writing, so I am familiar with them, and sometimes, they can give me a clue and help me fix errors.”

## Discussion

In the introduction of this paper, we stated that this study was guided by three related questions: the first question was to investigate EFL learners' preferences for WCF types regarding different types of errors; the second concerned the potential effects of proficiency and FLE level on their preferences for WCF types; and the third explored possible reasons they had for their preferences. Based on previous findings from the literature concerning perceptions of and preferences for WCF, we expected that our data would show that WCF preferences of learners in this study would have something to do with the nature of feedback itself and the type of error addressed. Overall, our expectations were confirmed—that is, while learners generally preferred more explicit types of WCF, the preference varied according to error type. In line with some earlier survey studies (e.g., Amrhein and Nassaji, 2010; Chen et al., 2016), our findings appear to mirror previous experimental studies reporting an advantage for more explicit types of WCF when focusing on specific linguistic errors (e.g., Bitchener, 2008; Guo and Barrot, 2019).

The finding that our participants were preferable for metalinguistic explanation for grammatical and orthographic errors is largely consistent with some previous studies (e.g., Karim and Nassaji, 2015). As mentioned in the literature, WCF can only be effective when learners understand the errors for which they received the WCF (Simard et al., 2015). According to Lee (2017), the type of metalinguistic explanation investigated in our study is considered highly explicit, which not only locates the error but provides detailed information about why it occurred and how it can be corrected. Responses from participants' interviews support this speculation, that is, the provision of metalinguistic explanation could enable them to notice and understand the errors, and more importantly, as in Bitchener and Knoch (2008, p. 415), it would allow them to “engage in guided learning and problem solving.”

Metalinguistic explanation was also viewed most useful for pragmatic errors by participants. To our best knowledge, few WCF studies have attempted to look into learners' perceived effectiveness of a WCF type for pragmatic errors, making a comparison of findings difficult. It is interesting, however, to note that this finding is similar to those found in several studies on oral error correction (e.g., Yang, 2016). Not surprisingly, pragmatic competence is acknowledged to be difficult for L2 learners to master (Rose, 2005), especially Thai EFL learners who appear to struggle with pragmatic transfer (Wannaruk, 2008). Thus, it is reasonable to assume that the participants were more likely to work out what the correction should be when receiving metalinguistic information about the sociolinguistic rules.

Despite participants' strong preference for metalinguistic explanation, almost all reported that they were rarely given it in class, which seems to suggest incongruence between teachers' WCF practices and students' preferences (see Li and Vuono, 2019), at least in the context of the present study. Lee (2017) explained that providing metalinguistic explanation is a time-consuming process and, therefore, not commonly employed by teachers in practice. To increase its practical value, researchers suggest students should be supplied with a metalinguistic explanation handout (as in Shintani and Ellis, 2013; Shintani et al., 2014).

There was a tendency for students to prefer overt correction to other three WCF types concerning lexical errors. Similarly, the learners in Bonilla López et al. (2018) favored overt correction over error code. One possible explanation for the finding can be found in Guo and Barrot (2019), in which learners found challenging to deal with item-based errors when metalinguistic explanation was provided; the difficulty might lie mainly in their lack of knowledge of the target feature. Lexical errors are idiosyncratic and involve item learning (Ellis, 2005); metalinguistic explanation or error code may help clarify the type of error (e.g., word choice) at best, but is not enough for learners to produce the correct form. As our interview results reveal, the participants at the low intermediate level expressed a similar concern that they would benefit more from overt correction. There is more evidence that participants' proficiency level may influence their preference for a more or less WCF type, which is discussed below.

Some researchers propose that less proficient learners require more explicit WCF assistance on less rule-governed, idiosyncratic linguistic errors than more proficient learners (Bitchener and Storch, 2016; Guo and Barrot, 2019). The present study supports this speculation. For all error types except for pragmatic ones, differences were found in preference for underlining and error code but not for overt correction and metalinguistic explanation between the two proficiency groups. One plausible interpretation here is that the upper intermediate learners might have greater metalinguistic awareness and sufficient knowledge about the target linguistic features and therefore were more likely to be prepared to respond to less explicit WCF type. The interview data reveal that the low English proficiency of learners at the low intermediate level might hinder their ability to self-correct lexical errors in response to underlining.

It worth noting that a recent study by Wei et al. (2020) reveals that L2 learners' writing proficiency has a negative association with most cases of L1-to-L2 rhetorical transfer, whereas their perception of L2 writing difficulty has a positive one. Despite that WCF was not a focus in their study, one possible implication can be made for understanding L2 learners' preferences for WCF types. That is, learners' L2 proficiency (or more specifically, L2 writing proficiency) may be related to their preferred WCF type for rhetorical errors. Moreover, the depth of L2 learners' processing of WCF influences their uptake of WCF, and successful uptake occurs only if they notice and understand the intention of WCF provided by teachers (Lee, 2017). It would be interesting to look into whether L2 proficiency and foreign language enjoyment are two learner variables that affect noticing and understanding rhetorical errors identified by WCF. However, this type of errors was not examined in our study, so that it is not possible to extend our discussion on these issues, which are definitely worth studying further.

There is also evidence of differences in preference by low and high FLE groups. The finding that high FLE learners perceived both less explicit WCF types—error code and underlining—as useful is supported by previous studies. FLE is linked to learners' pride in achieving something difficult (Dewaele and MacIntyre, 2016) and takes on dimensions such as intellectual focus, heightened attention, and optimal challenge (Boudreau et al., 2018). It is reasonable to assume that learners with more FLE are more motivated to take up the challenges posed by teachers' provision of less explicit WCF types. In other words, they are more willing to engage in self-correction. In addition, high FLE students also concerned teachers' workload, as suggested by the qualitative data. As argued by Dewaele and MacIntyre (2019), FLE is mostly triggered by teacher-related variables such as attitudes toward the teacher and teacher friendliness. High FLE learners usually keep good relationships with teachers and may try to be considerate to decrease teachers' workload by engaging in self-correction. Moreover, the finding that error code was rated higher by high FLE learners than low FLE learners at the lower proficiency level could be explained by the mediating effects of FLE on learner-related variables such as proficiency (Zhang et al., 2020). In other words, just like upper intermediate learners, learners with lower level of proficiency also preferred less explicit WCF types when they scored high on FLE scales. Our study highlights the positive influences of FLE on the preference for WCF types, and by extension, L2 writing.

Unlike the previous studies, which generally reported that learners tended to show a preference for comprehensive WCF (e.g., Lee, 2005; Amrhein and Nassaji, 2010), students in this study were divided in their preference for selective or comprehensive WCF. A key contributor to this result was the participants' FLE level. It is possible that learners with high FLE were more willing to be engaged with self-correction with selective WCF. This is echoed by Chen et al. (2016, p. 9) as their study found that students who preferred selective WCF expected “some degree of independence in their revision processes.”

## Conclusion

In addition to providing insight into learners' preferences for WCF types on specific errors in the Thai EFL context, this study is the first to show interactive effects of proficiency and FLE level on such preferences. The results of the study were complex, but a clear picture emerged. Learners at the upper intermediate level found underlining and error code to some degree useful, but the low intermediate learners did not, while the two proficiency groups preferred metalinguistic explanation for most error types and overt correction for lexical errors. Results also provided evidence for the role of FLE in influencing individuals to perceive the value of less explicit WCF types as well as selective vs. comprehensive WCF. Although it is difficult to make any direct pedagogical implications based on a single study, this study's findings may encourage Thai EFL teachers, and teachers in similar EFL contexts, to provide more explicit types of WCF on learners' errors in writing. Teachers should also feel confident in using a selective WCF approach to motivate learners to be engaged with WCF, especially those with high FLE. This study opens up an avenue toward understanding how and why FLE may come to play a role in WCF instruction.

Several limitations need to be noted when interpreting the findings of this study. First, the study sample was drawn from a population of students at a large private university where English is used as the medium of instruction. Thus, the findings may have direct relevance uniquely for, but limited to, the EAP program at the university or other local universities with a similar institutional context. Second, as this study only involved low and upper intermediate students, some of the findings may not portray the learning situation of students at other levels. The sample size for the interview was relatively small; to make the sample more representative of the population, the number of participants would have had to be larger. Another limitation is the absence of consideration of teacher variables. As mentioned earlier, the divergence between students' and teachers' preferences for WCF types can lead to inefficient WCF instruction, suggesting that teachers' practice of WCF needs to be considered as it may affect or shape students' perceptions of the usefulness of WCF. More interestingly, one would expect that teacher variables such as WCF knowledge or charisma may have something to do with students' FLE, which, in turn, influences their perceptions toward WCF. Thus, as Cheng and Zhang (2021) suggest, future research on WCF can benefit from examining teacher variables. Furthermore, although the WCFPQ items used in this study developed based on learners' real practices in the classroom, only two error examples were used for each linguistic aspect, making it impossible to reach a firm conclusion about learners' preferred WCF types on other errors. For instance, Zhang (2018) showed that metalinguistic explanation was considered ineffective for irregular past tense forms (which were not included in the WCFPQ) by low intermediate EFL learners. It is hoped that future research will continue to shed light on this research area with different institutional contexts, and by using a larger sample and error resources of different varieties.

## Data Availability Statement

The original contributions presented in the study are included in the article/Supplementary Material, further inquiries can be directed to the corresponding author/s.

## Ethics Statement

The studies involving human participants were reviewed and approved by the Ethics Committee of University of Electronic Science and Technology of China. The patients/participants provided their written informed consent to participate in this study.

## Author Contributions

TZ and XC conceived and planned the research project. TZ and PK carried out the data collection and analysis. TZ, XC, and JH contributed to the interpretation of the results. TZ took the lead in writing the manuscript. All authors provided critical feedback and helped shape the research, analysis, and manuscript.

## Conflict of Interest

The authors declare that the research was conducted in the absence of any commercial or financial relationships that could be construed as a potential conflict of interest.

## Footnotes

^1^The learners in their studies were also asked to rate the usefulness of WCF on content and organization errors. Considering that WCF is generally defined as responses to linguistic-related errors to facilitate improved writing accuracy, we did not discuss these two types of errors in this study.

^2^Students are exempted from the English course(s) based on their IELTS scores. For example, to be exempted from attending English I, students need to achieve an overall band score of 5.

^3^This study did not involve students from English I and English II classes as they were intermediate learners. A larger language proficiency gap (low vs. upper) might be expected to produce greater differences.

^4^The choices were made based on a list of the most common error instances in students' writing samples.
