# Supplementary material for: EFL Students' Preferences for Written Corrective Feedback: Do Error Types, Language Proficiency, and Foreign Language Enjoyment Matter?
Source: Front Psychol. 2021 Apr 8;12:660564. doi: 10.3389/fpsyg.2021.660564 (PMC8060554; doi:10.3389/fpsyg.2021.660564)
Supplement: Supplementary file 1 [file Data_Sheet_1.pdf]

Appendix 1

Written Corrective Feedback Preference Questionnaire

The purpose of this survey is to help us discover more effective ways of providing corrective feedback on learning English. Your voices are important to us. Please answer the questions below. This survey will take approximately 10 min to complete. Your participation in this survey is entirely voluntary, and your responses will be kept confidential and anonymous.

Section A: Personal background information

1. Name：

____

2. Gender：

○Male ○Female

3. Age:

____

4. How long have you been studying English?

____

5. Which English course are you enrolled in at present?

○Basic

○English I

○English II

○English III

○English IV

○I have completed English IV

6. Have you taken an IELTS? If yes, what is your score?

○No, I haven't.

○Below 5

○5

○5.5

○6

○above 6

Section B: Preferences for written corrective feedback in the classroom.

Please tell me to what extent you like or dislike the following written feedback that teachers provide in writing.

[Not at all useful = 1; Not very useful = 2; Somewhat useful = 3; very useful =4; extremely useful =5]

Error example 1:

A student wrote the following sentence in a story:

This fox got some apples and droped them into the bag.

1. The teacher first underlines the error and then gives its correct form: This fox got some grapes and droped [dropped] them into the bag.

Not at all useful	Not very useful	Somewhat useful	Very useful	Extremely useful

2. The teacher just underlines the error: This fox got some apples and droped them into a bag

Not at all useful	Not very useful	Somewhat useful	Very useful	Extremely useful

3. The teacher underlines the error and explains why it was an error: This fox got some apples and droped them into a bag. (the past tense form of drop is wrong. You need to double the ending consonant letter and add –ed if a verb ends in a vowel and a consonant. For example, stop–stopped.)

Not at all useful	Not very useful	Somewhat useful	Very useful	Extremely useful

4. The teacher uses a symbol (VF = verb form) to indicate the nature of the error: This fox got some apples and droped (RPT) them into a bag.

Not at all useful	Not very useful	Somewhat useful	Very useful	Extremely useful

Error example 2–A student wrote the following sentence to describe his teacher*:*
That is our respectful teacher.

5. The teacher just underlines the error: That is our respectful teacher.

Not at all useful	Not very useful	Somewhat useful	Very useful	Extremely useful

6. The teacher underlines the error and explains why it was an error: That is our respectful teacher. (It is a word choice error in this context. Consider an alternative word.)

Not at all useful	Not very useful	Somewhat useful	Very useful	Extremely useful

7. The teacher uses a symbol (WW = wrong word) to indicate the nature of the error: That is our respectful(WW) teacher.

Not at all useful	Not very useful	Somewhat useful	Very useful	Extremely useful

8. The teacher first underlines the error and then gives its correct form: That is our respectful [respectable] teacher

Not at all useful	Not very useful	Somewhat useful	Very useful	Extremely useful

Error example 3–A student wrote the following sentence in his essay*:*

All kinds of types of colours are beautiful.

9. The teacher just underlines the error: All kinds of types of colours are beautiful.

Not at all useful	Not very useful	Somewhat useful	Very useful	Extremely useful

10. The teacher underlines the error and explains why it was an error: All kinds of types of colours are beautiful. (A redundancy error occurs in this sentence because the two phrases *kinds of* and *types of* express the same meaning.)

Not at all useful	Not very useful	Somewhat useful	Very useful	Extremely useful

11. The teacher uses a symbol (WW = wrong word) to indicate the nature of the error: All kinds of types of (WW) colours are beautiful.

Not at all useful	Not very useful	Somewhat useful	Very useful	Extremely useful

12. The teacher corrects the error by deleting it: All ~~kinds of~~ types of colours are beautiful.

Not at all useful	Not very useful	Somewhat useful	Very useful	Extremely useful

Error example 4: A student wrote the following sentence in his essay:

Visitors can get to the grand palace by boat.

13. The teacher underlines the error and explains why it is an error: Visitors can get to the grand palace by boat. (Capitalize names and other proper nouns, for example, the name of a city such as Bangkok).

Not at all useful	Not very useful	Somewhat useful	Very useful	Extremely useful

14. The teacher uses a symbol (Cap = capitalization) to indicate the nature of the error: Visitors can get to the grand(Cap) palace(Cap) by boat.

Not at all useful	Not very useful	Somewhat useful	Very useful	Extremely useful

15. The teacher just underlines the error: Visitors can get to the grand palace by boat.

Not at all useful	Not very useful	Somewhat useful	Very useful	Extremely useful

16. The teacher first underlines the error and then gives its correct form: Visitors can get to the grand palace Grand Palace by boat.

Not at all useful	Not very useful	Somewhat useful	Very useful	Extremely useful

Error example 5: A student wrote a sentence in an email which is sent to an elder friend from Australia:

Can you provide any advice for a student like me who wants to speak English better?

17. The teacher first underlines the error and then gives its correct form: Can [Could] you provide any advice for a student like me who wants to speak English better?

Not at all useful	Not very useful	Somewhat useful	Very useful	Extremely useful

18. The teacher underlines the error and explains why it is an error: Can you provide any advice for a student like me who wants to speak English better? (the modal verb "can" is a tone error because it is not polite when writing an email to elders or seniors.)

Not at all useful	Not very useful	Somewhat useful	Very useful	Extremely useful

19. The teacher uses a symbol (PF = pragmatic failure) to indicate the nature of the error: Can$^{(PF)}$ you provide any advice for a student like me who wants to speak English better?

Not at all useful	Not very useful	Somewhat useful	Very useful	Extremely useful

20. The teacher just underlines the error: Can you provide any advice for a student like me who wants to speak English better?

Not at all useful	Not very useful	Somewhat useful	Very useful	Extremely useful

Error example 6–A student wrote the following sentence using the word *near:*

There is the park near my home.

21. The teacher underlines the error and explains why it was an error: There is the park near my home (When you mention an object for the first time, you use *a* or *an*.)

Not at all useful	Not very useful	Somewhat useful	Very useful	Extremely useful

22. The teacher underlines the error: There is the park near my home.

Not at all useful	Not very useful	Somewhat useful	Very useful	Extremely useful

23. The teacher first underlines the error and then gives its correct form: There is the [a] park near my home.

Not at all useful	Not very useful	Somewhat useful	Very useful	Extremely useful

24. The teacher uses a symbol (Art = article) to indicate the nature of the error: There is the[(ART)] park near my home.

Not at all useful	Not very useful	Somewhat useful	Very useful	Extremely useful

Error example 7–A student wrote the following sentence in a request letter to the university administrator*:*

I want your answer to my question.

25. The teacher underlines the error: I want your answer to my question.

Not at all useful	Not very useful	Somewhat useful	Very useful	Extremely useful

26. The teacher underlines the error and explains why it was an error: I want your answer to my question. (The sentence is grammatically correct, but the use of *want* is very demanding and is not appropriate when requesting information. Consider how to soften the tone of the request.)

Not at all useful	Not very useful	Somewhat useful	Very useful	Extremely useful

27. The teacher uses a symbol (PF = pragmatic failure) to indicate the nature of the error: I want[(PF)] your answer to my question.

Not at all useful	Not very useful	Somewhat useful	Very useful	Extremely useful

28. The teacher corrects the error by deleting it: I want [would like to have] your answer to my question.

Not at all useful	Not very useful	Somewhat useful	Very useful	Extremely useful

Error example 8–A student wrote the following sentence in his news report*:*
Apple will announce it's new iWatch.

29. The teacher just underlines the error: Apple will announce it's new iWatch.

Not at all useful	Not very useful	Somewhat useful	Very useful	Extremely useful

30. The teacher underlines the error and explains why it was an error: Apple will announce it's new iWatch. (*It's* is a contraction for *it is*. Consider whether an apostrophe ['] is needed here.)

Not at all useful	Not very useful	Somewhat useful	Very useful	Extremely useful

31. The teacher uses a symbol (P = punctuation unnecessary) to indicate the nature of the error: Apple will announce it's $^{(P)}$ new iWatch.

Not at all useful	Not very useful	Somewhat useful	Very useful	Extremely useful

32. The teacher corrects the error by deleting it: Apple will announce it's [its] new iWatch

Not at all useful	Not very useful	Somewhat useful	Very useful	Extremely useful

Section C: To what extent do you agree with the following statement?

[Strongly disagree = 1; Disagree = 2; Undecided =3; Agree = 3; Strongly agree = 5]

Teachers should correct all errors, major and minor.

Strongly disagree	Disagree	Not sure	Agree	Strongly agree

You have answered all questions. Please submit and thank you.
