# Supplementary material for: EFL Students' Preferences for Written Corrective Feedback: Do Error Types, Language Proficiency, and Foreign Language Enjoyment Matter?
Source: Front Psychol. 2021 Apr 8;12:660564. doi: 10.3389/fpsyg.2021.660564 (PMC8060554; doi:10.3389/fpsyg.2021.660564)
Supplement: Supplementary file 2 [file Data_Sheet_2.pdf]

Appendix 2

Reliability Analysis for Foreign Language Enjoyment Scale

Item		Corrected item–total correlation	Cronbach alpha if item deleted
1	I can be creative in learning English	.448	.832
2	I can laugh off embarrassing mistakes in English writing	.323	.840
3	I don't get bored of learning English	.312	.844
4	I enjoy English.	.451	.831
5	I often feel happy during the English class	.408	.833
6	I learnt to express myself better in English	.433	.832
7	I'm a worthy member of the English class	.519	. 828
8	I've learnt interesting things by learning English	.448	.831
9	In class, I feel proud of my accomplishments	.477	.829
10	It's a positive environment	.533	.828
11	It's cool to know English	.341	.843
12	English's fun	.317	.838
13	Making errors is part of the learning process	.332	.838
14	The classmates are nice	.375	.834
15	The teacher is encouraging	.463	.830
16	The teacher is friendly	.463	.830
17	The teacher is supportive	.428	.832
18	There is a good atmosphere	.422	.832
19	We form a tight group	.506	.829

20	My English class is active, for example, we are running jokes.	.637	.822
21	We laugh a lot in English classes.	.502	.829
